# Psychosocial Consequences of Excess Weight and the Importance of Physical Activity in Combating Obesity in Children and Adolescents: A Pilot Study

**DOI:** 10.3390/nu17101690

**Published:** 2025-05-15

**Authors:** Małgorzata Wąsacz, Izabela Sarzyńska, Danuta Ochojska, Joanna Błajda, Oliwia Bartkowska, Karolina Brydak, Szymon Stańczyk, Martyna Bator, Marta Kopańska

**Affiliations:** 1 Department of Medical Psychology, Faculty of Medicine, University of Rzeszów, 35-959 Rzeszów, Poland; mwasacz@ur.edu.pl; 2 Student Research Club “Reh-Tech”, Faculty of Medicine, University of Rzeszów, 35-959 Rzeszów, Poland; sarzynskaizabela-2001@wp.pl (I.S.); ob130723@stud.ur.edu.pl (O.B.); kb121609@stud.ur.edu.pl (K.B.); ss131630@stud.ur.edu.pl (S.S.); mb135954@stud.ur.edu.pl (M.B.); 3 Faculty of Health Sciences and Psychology, University of Rzeszów, 35-959 Rzeszów, Poland; dochojska@ur.edu.pl (D.O.); jblajda@ur.edu.pl (J.B.)

**Keywords:** overweight, obesity, children, adolescents, physical activity, self-esteem, mental health

## Abstract

**Introduction:** Overweight and obesity among children and adolescents are a growing public health problem. The aim of this study was to determine the impact of overweight and obesity on the psychosocial functioning and physical activity levels of children and adolescents. **Methods:** This study was conducted among 100 children and adolescents aged 9–18 from three institutions in the Podkarpackie Province: a primary school, a hospital ward, and a health resort. An original questionnaire was used, containing questions about body image, emotions related to eating, self-esteem, and physical activity. All participants were classified into appropriate BMI categories based on anthropometric data. **Results:** The analysis showed that 48% of respondents admitted to eating meals in secret, and 5% did so every day for a month. As many as 65.82% of participants stated that their body shape affects the way they think about themselves, and more than two-thirds felt frustrated with their figure. Statistically significant differences were observed in the perception of one’s own body and the level of its acceptance depending on the BMI category. In addition, 58.58% of respondents did not engage in physical activity to control their weight. **Conclusions:** The results of this study confirm that overweight and obesity in children and adolescents are associated with a negative body image, low self-esteem, unhealthy eating habits, and low levels of physical activity. The obtained results can be used by medical personnel, educators, and educational institutions to develop effective prevention programs aimed at counteracting the negative consequences of overweight and obesity in children and adolescents.

## 1. Introduction

A healthy, balanced diet plays a key role in the health and proper functioning of the body, both physically and mentally, in every human being. Children and young people are a special group whose eating habits have an impact on their health in adulthood. Regular physical activity is also an important element of the proper development of the younger generation, as it has a positive impact on the functioning of the body. Low levels of physical activity, a sedentary lifestyle, and sleepiness play a key role in the development of obesity [1,2]. Currently, the prevalence of overweight children and adolescents is increasing dramatically, which can lead to adverse health consequences such as type II diabetes, hypertension, and obstructive sleep apnoea. In addition, overweight and obesity can contribute to negative consequences in the psychosocial functioning of young people [3].

An increase in the number of cases of overweight and obesity is observed almost worldwide, leading to alarming statistics and forcing action to be taken at various levels, from family to international [4,5,6]. In terms of physical health, overweight and obesity increase the risk of many chronic diseases, such as type 2 diabetes, hypertension, cardiovascular disease, and musculoskeletal problems. These problems can appear as early as childhood, but most often become more severe in adulthood, leading to premature death and a reduced quality of life [7].

The psychological aspects should not be forgotten either. Overweight or obese children and adolescents often struggle with low self-esteem, depression, and social isolation, which negatively affects their emotional and social development. The stigma associated with being overweight can lead to difficulties in peer relationships and learning problems. The causes of overweight and obesity are multifactorial and include both genetic predisposition and environmental factors such as poor eating habits, physical inactivity, and the influence of the media and advertizing. The role of the family, school, and the wider social environment is crucial in shaping healthy habits and preventing overweight and obesity [8].

Body image plays a crucial role in the psychosocial development of children and adolescents. Negative body image is frequently associated with low self-esteem, unhealthy eating behaviours, and reduced physical activity, all of which contribute to the development and maintenance of overweight and obesity. Exposure to unrealistic body ideals through media and peer comparisons exacerbates these issues, particularly during adolescence, a period characterized by increased sensitivity to appearance-related concerns [9].

Obesity is a complex health problem that can be divided into two main categories: simple obesity and secondary obesity. In most cases, children and adolescents are affected by simple obesity, the main cause of which is a positive energy balance—consuming more calories than are burned. Simple obesity is the most common form of obesity in children and adolescents. One of the key causes of this form of obesity is excessive calorie intake, which is directly related to an inadequate diet. Ready meals and fast food, which contain large amounts of sugar and saturated fats and are low in vitamins and micronutrients, promote the growth of adipose tissue. In addition, irregular meals and excessive portions also contribute to overweight and obesity. Children often eat too much and too often, which leads to a calorie surplus [10,11].

Another important factor is physical inactivity. Modern children and young people spend a lot of time watching television, playing computer games, and using social media. These types of entertainment are replacing traditional forms of physical activity such as walking, cycling, and playing games. In addition, fewer children spend time playing actively with their peers, as most interactions take place online. A sedentary lifestyle accompanies children from an early age, leading to gradual weight gain. Poor family relationships also influence the development of obesity in children and adolescents. Children who experience difficult family situations, such as parental divorce, sexual abuse, or excessive demands, often seek comfort in food. In such cases, eating becomes a mechanism for coping with stress, leading to eating disorders and weight gain. Children may treat food as a reward for surviving stressful situations, which further disrupts their healthy eating habits [12]. It should be noted that in recent years, the problem of overweight and obesity in children in Poland has become the subject of numerous studies and analyses. One of the main factors influencing abnormal body weight in children is poor eating habits shaped by their parents. Parents often do not control how much time their children spend in front of TV screens, computers, or phones, which limits their physical activity. In addition, some parents excuse their children from physical education classes, which is a mistake, as exercise is crucial for maintaining a healthy weight [13].

Solving this problem requires integrated action on many levels, from health education and the creation of conditions conducive to physical activity to appropriate nutritional and medical policies. Only through coordinated efforts is it possible to halt the increase in the number of overweight and obese children and adolescents and improve the health of future generations [5,6].

Physical activity plays an important role in prevention and weight loss, enabling increased energy expenditure and supporting the maintenance of healthy eating habits [14,15]. What is more, regular exercise brings a number of health benefits, including improved cardiovascular function, support for the development of the musculoskeletal system, and a positive impact on the mental health and self-esteem of children and young people [16,17,18]. Engagement in physical activity often becomes an important motivator for young people to change their lifestyle and strive to achieve or maintain a slim figure. Participation in sports activities not only improves physical fitness but also builds a sense of effectiveness and belief in one’s own abilities, which strengthens the determination to continue health-promoting activities [19,20]. It is worth noting that various educational programmes implemented in schools, such as SPARK and Planet Health, have proven highly effective in increasing the level of physical activity among students and reducing the incidence of overweight and obesity [21,22]. The influence of the social environment is also significant. Support from family and peers, as well as favourable environmental conditions, can clearly encourage children and young people to be more physically active. The presence of positive role models and joint activities with loved ones help to reinforce healthy habits [23,24]. Furthermore, especially during adolescence, the motivation to participate in sports often stems from the need to improve physical appearance, which further encourages young people to take action to reduce their body weight [25]. In view of the growing problem of low physical activity, increasingly referred to as a pandemic, it is important to take comprehensive measures to promote physical activity among children and adolescents. Physical activity can not only support weight loss but also be a key motivating factor for making lasting lifestyle changes [26,27].

Physical activity brings numerous health benefits in physical, mental, and social dimensions. Regular exercise improves cardiovascular fitness, strengthens muscles and bones, positively influences mood, and enhances self-esteem. The World Health Organization (WHO) recommends that children and adolescents aged 5–17 engage in at least 60 min of moderate or vigorous physical activity daily [28]. According to the Active Healthy Kids Poland 2022 report, most physical activity indicators for children and adolescents in Poland received moderate or poor ratings (from C+ to D), and two key areas—overall physical activity and active play—were assessed as incomplete due to a lack of data. These trends are consistent with global data, which show that more than 80% of adolescents worldwide do not meet WHO recommendations [29]. These patterns highlight the need to analyse psychosocial and behavioural determinants of physical inactivity, particularly among adolescents with excessive body weight. This article aims to determine the impact of overweight and obesity on the psychosocial functioning and physical activity levels of children and adolescents. In the face of the growing prevalence of obesity among children and adolescents, the development of effective, multi-faceted prevention programs that address both the physical and mental health aspects of young people is becoming increasingly important. The obtained results provide valuable insights into the need for designing interventions that include nutritional counselling, the promotion of regular physical activity, and psychological support based on scientific evidence, aimed at improving body image and self-esteem [3]. The involvement of families and local stakeholders is crucial in strengthening positive health habits and creating a supportive environment that fosters long-term behavioural changes. At the same time, prevention strategies must address the widespread influence of digital media, which perpetuates unrealistic beauty standards, contributing to the deterioration of mental health and the development of maladaptive coping mechanisms [30]. Effective programs should be based on proven theories, such as the Health Belief Model and the Self-Determination Theory, which support lasting pro-health attitudes and behaviours [31].

It is worth noting that this study is not a direct continuation of previous research but aims to deepen the understanding of the relationship between BMI and the psychosocial functioning of children and adolescents (including body image, self-esteem, and motivation), taking into account different types of institutions, which has rarely been analysed so far. Although the general consequences of overweight and obesity are well known, there is still a lack of data on how specific BMI categories affect motivation for physical activity and self-esteem in children and adolescents. A better understanding of these relationships will help develop more effective preventive and therapeutic strategies.

## 2. Materials and Methods

### 2.1. Participants and Recruitment Process

A total of 100 respondents from three units in south-eastern Poland took part in the study: the Health Resort Hospital (n = 31), the provincial clinical hospital (n = 31), and the General Secondary School (n = 38). Although 110 individuals were initially invited to participate in this study, 7 did not provide consent or withdrew before completing the questionnaire. Additionally, 3 questionnaires were excluded due to missing data, which made it impossible to calculate BMI or perform psychometric analysis. As a result, the final study sample consisted of 100 participants. The research group was diverse in terms of gender, age, body weight, height, BMI, and place of residence. In terms of sex distribution, girls made up 54% of the sample and boys 46%. The age range of the respondents was 9 to 18 years. An analysis of place of residence showed that 61% of respondents came from cities and 39% from rural areas. In summary, the study group was well diversified and representative of young people from different backgrounds and institutions. The greatest diversity in health parameters was observed at the Health Resort Hospital, while the most homogeneous group was high school students. The sample was selected deliberately and in cooperation with three educational and medical institutions located in the Podkarpackie Province. The study participants were recruited from three different types of institutions based on their specific characteristics and the potential to obtain representative data on eating habits, health attitudes, and motivation for weight loss among children and adolescents. Each of these institutions represented different age and health groups, allowing for a diverse analysis of the results. The secondary school was selected due to its access to students at an age when eating habits and motivation for weight control are being formed. The clinical hospital ward provided data on patients with chronic conditions related to obesity and metabolic diseases, enabling analysis of how these illnesses influence dietary attitudes and motivation for a healthy lifestyle. In turn, the health resort hospital allowed for the collection of information on the impact of a health-promoting environment on patients’ health and motivation in the weight loss process. The sample was selected using purposive sampling, as each institution represented a distinct group of participants, which made it possible to gather data from various perspectives on eating habits, weight loss motivation, and the influence of health status on these attitudes. Participants were contacted directly through the institutions they attended or where they were staying for treatment or rehabilitation. Participation in this study was entirely voluntary. Inclusion criteria included age between 9 and 18 years, the ability to complete the questionnaire, and the provision of informed consent. Children with cognitive or physical limitations that prevented questionnaire participation were excluded. Consent was obtained from the management of the institutions, and then information activities were carried out aimed at potential participants and their parents or legal guardians. Written informed consent to participate in the study was obtained from all participants aged 16 to 18 years. In the case of younger children (under 16 years of age), written consent was provided by their parents or legal guardians, and the children themselves gave verbal assent to participate in this study.

This study was cross-sectional and was carried out in 2023–2024. Respondents completed an anonymous questionnaire and underwent basic anthropometric measurements (weight, height), on the basis of which BMI was calculated. Recruitment, data collection, and analysis were carried out in accordance with applicable ethical standards. The study design was approved by the Bioethics Committee of the District Medical Chamber in Rzeszów (78/2022/B). Informed consent to participate in this study was obtained from all respondents (or their legal guardians).

### 2.2. Research Questionnaire and Data Collection Procedures

All participants completed a proprietary Child Nutrition Questionnaire, which was one of the basic research tools used in this project. This tool was developed specifically for this study and based on concepts related to nutritional behaviours and body image assessment. The form was anonymous and single-use, and its purpose was to collect data on eating habits, body image, attempts to control weight, and emotions associated with eating. It is worth noting that anthropometric measurements were conducted in person, but no data allowing the identification of participants were recorded, including questionnaire results and body measurements. The data collected through the questionnaires were anonymized after collection, and body measurements were coded using individual ID numbers without any identifying information. The database did not store names, surnames, or any other personal data that could allow participant identification. All data were anonymized at the stage of entering them into the research database, which prevents the identification of individuals in subsequent analyses. The questionnaire included questions about, among other things, the frequency of overeating, dietary restrictions, body image concerns, physical activity undertaken to control weight, as well as self-perception and body image. The language of the questionnaire was adapted to be age-appropriate and understandable for children and adolescents. For younger participants, simplified explanations of selected terms were provided if necessary. If participants encountered difficulties in understanding certain items, trained research assistants or parents provided clarification, particularly for children under the age of 12.

The measurements were carried out by members of the research team who had been properly prepared and trained. The measurements were taken using standardized equipment in conditions ensuring the comfort and privacy of the participants, following uniform procedures regardless of the location, whether in a hospital or an educational facility. Measurement data were recorded immediately after each measurement on paper coding sheets that contained only the participant’s individual ID number, without any personal information.

After the completion of the measurement process, the sheets were handed over to a designated research team member responsible for data digitization. Data entry into the database was performed manually. The database was properly secured and accessible only to authorized members of the research team. The data recording and entry process was designed to prevent participant identification while ensuring the consistency and reliability of the collected information.

The collected responses were used to assess eating behaviours and their potential links to overweight, obesity, and motivational and psychosocial factors in children and adolescents. This tool provided a detailed picture of the respondents’ relationship with food and their own bodies. Selected elements of Self-Determination Theory (SDT) by Deci and Ryan were used to assess the level of motivation to reduce body weight, taking into account both internal and external motivation as well as lack of motivation (amotivation [32]. Reference was also made to the Stages of Change Model, which allows the level of participants’ readiness to act to be determined. The average time required to complete the questionnaire was approximately 15–20 min, ensuring that participants remained engaged and attentive throughout the process.

### 2.3. Measurements and BMI Classification

In order to obtain empirical data, a questionnaire developed specifically for this study was used, which contained closed-ended questions. The questionnaire was supplemented by anthropometric measurements taken in counselling and school settings. Height was measured to the nearest 0.1 cm using a SECA 213 height gauge, and weight was measured using a SECA 813 electronic scale (accuracy 0.1 kg). The measurements were taken in light clothing, without shoes, in accordance with standardization rules—each value was recorded twice and the average value was used for analysis. On this basis, the BMI (Body Mass Index) was calculated. For BMI classification, age- and sex-specific percentile charts developed by the World Health Organization (WHO) were used for participants under 18 years of age. For adolescents aged 18 years, adult BMI categories defined by WHO were applied. In addition, in order to obtain a more in-depth picture of psychosocial factors, individual interviews were conducted with a selected subgroup of respondents (n = 50) whose results indicated a particularly low or high level of motivation to change their body weight.

### 2.4. Statistical Analysis Methods

The collected data were analysed using IBM SPSS Statistics (version 27.0) and Microsoft Excel 365. Descriptive statistics were first used to present the characteristics of the study group, including calculations of means, medians, standard deviations, and value ranges and frequencies for categorical data. To compare differences between groups based on gender, place of residence, or BMI category, appropriate inferential statistical tests were applied. Pearson’s chi-square test was used to analyse qualitative variables. For continuous variables, the Student’s *t*-test for independent samples was used if normal distribution assumptions were met, and the Mann–Whitney U test was used when those assumptions were not fulfilled. The analysis focused primarily on descriptive statistics and group comparisons using chi-squared tests and non-parametric methods.

The internal consistency of the measurement tools, including scales assessing motivation and health-related behaviours, was tested using Cronbach’s alpha coefficient. The reliability of the instruments was confirmed, with alpha values ranging from 0.72 to 0.84. In instances where answers were missing—never exceeding 3% within any dataset—listwise deletion was used to preserve the overall integrity of the data. All statistical tests were conducted using a significance level of α = 0.05, with p-values below 0.05 considered statistically significant. Where appropriate, measures of effect size, such as Cramér’s V or eta-squared, were also calculated to provide context for the practical importance of the results. This comprehensive statistical approach aimed not only to test group-level differences but also to explore complex relationships between body weight, physical activity, self-perception, and psychosocial variables across a diverse adolescent population.

## 3. Results

### 3.1. Basic Characteristics of the Population Covered by This Study

An analysis of the structure of the study group (N = 100) revealed clear differences between the three units in terms of demographic and anthropometric characteristics. The largest differences were mainly in body weight, height, and BMI distribution, which reflect both the specific nature of the institutions and the diverse health needs of their residents. Despite a relatively even gender distribution in the entire sample (55% girls vs. 45% boys), in individual terms, there was a predominance of girls in educational (Tyczyn) and health resort (Zimowit) facilities, while in the clinical hospital (Rzeszów), the gender ratio was more balanced, with a slight predominance of boys. In terms of average age, the group from the General Secondary School stood out clearly—it was homogeneous in age, with a predominance of 18-year-olds. In contrast, the Health Resort and Provincial Clinical Hospital had a wider age range (9–18 years), which may be due to the openness of these facilities to younger patients, who are often undergoing treatment or rehabilitation. Respondents from Rymanów-Zdrój differed significantly from the others—their body weight was on average more than 16 kg higher than that of young people from the secondary school. The standard deviation in this group was also the highest, which indicates a high degree of heterogeneity in terms of weight and probably the presence of both underweight and significantly obese individuals. The highest height was achieved by participants from Tyczyn, who also showed the least variation in this trait, which may indicate a relatively homogeneous lifestyle and level of physical development in this population. In the other two groups (hospitalized), the distribution of height was more dispersed, which may be the result of health or age differences. Analysis of the BMI index allowed three distinct profiles of individuals to be identified (Table 1):

- Zimowit: greatest diversity, presence of cases of obesity I and II, high percentage of overweight and obese people (22 people in total);
- Rzeszów: moderate diversity, predominance of overweight (10 people) and normal weight, presence of obesity I and II;
- Tyczyn: healthiest weight profile—no obesity, predominance of normal body weight (55%), limited number of cases of underweight and overweight.

### 3.2. Dietary Behaviour and Physical Activity Related to Weight Control

#### 3.2.1. Eating in Secret

**Figure 1:**
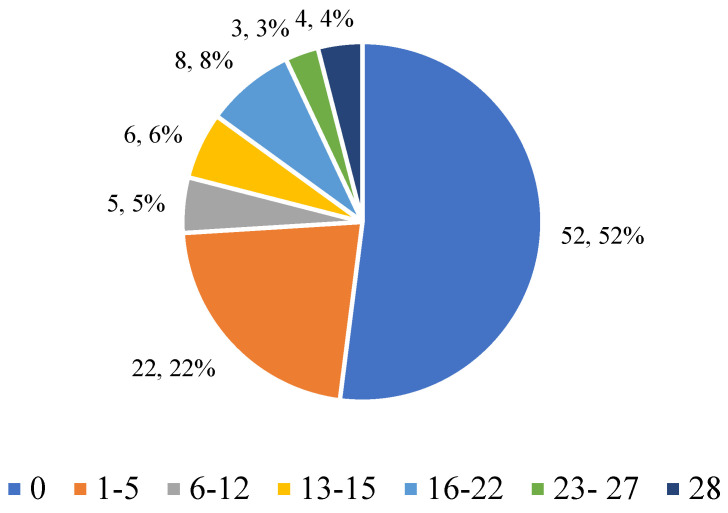
Respondents’ answers to the question “Have you ever eaten in secret?”.

In order to assess the extent of secret eating, participants were asked how many days in the last month they had eaten meals in secret (Figure 1). The majority of respondents (n = 52; 65.82%) declared that they had never engaged in such behaviour, which may indicate a healthy relationship with food. Sporadic cases (1–5 days) were reported by 27.85% of respondents (n = 22), while regular or intense food hiding (more than 6 days) was reported by approximately 15% of participants. Particularly worrying were the responses of 4 people (5.06%) who declared that they ate in secret for the entire month. Although this problem did not affect the majority, the data indicate the presence of risky behaviours in part of the group, which may require further psychodietetic assessment.

#### 3.2.2. The Desire to Have a Flat Stomach

**Figure 2:**
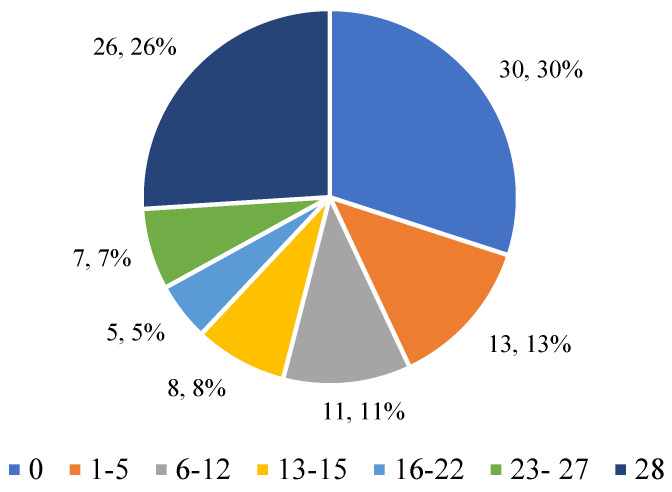
Respondents’ answers to the question “Did you want your stomach to be completely flat?”.

Figure 2 illustrates how often respondents felt the desire to have a completely flat stomach during the last month. The largest group (37.97%) said they never felt this way, which may indicate less pressure to look good in this group. On the other hand, 32.91% of respondents said they thought about having a flat stomach every day for 28 days, suggesting significant pressure related to body image. The remaining responses ranged from sporadic (1–5 days) to regular occurrence of this desire (up to 27 days), indicating varying levels of commitment to changing one’s figure.

#### 3.2.3. The Influence of Body Shape on Self-Perception

**Figure 3:**
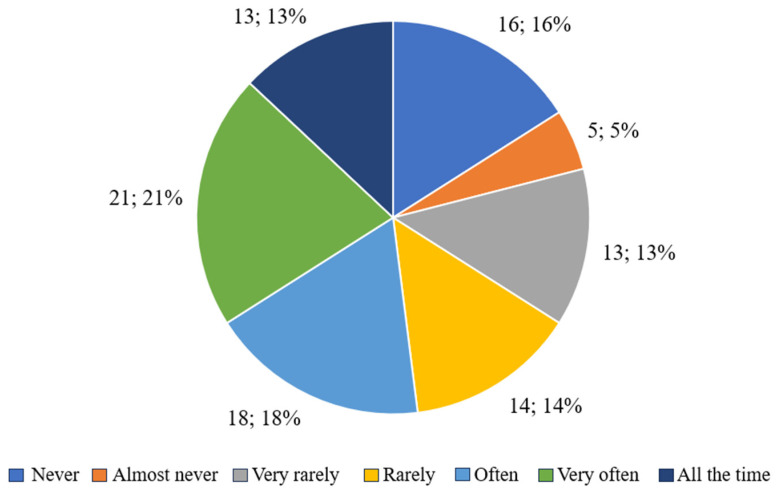
Respondents’ answers to the question “Has your body shape affected the way you think about yourself?”.

Figure 3 shows the distribution of respondents’ answers to the question concerning the impact of perceived body shapes on the way they think about themselves, which allows for an assessment of the strength of the relationship between body image and self-esteem.

The results show that 65.82% of respondents declared that their body shape significantly affects how they feel about themselves, indicating answers ranging from “often” to “all the time.” In particular, 21 people (26.58%) selected “very often” and 13 respondents (16.46%) selected “all the time”, which may indicate a strong link between body shape and identity and self-esteem. In addition, 18 people (22.78%) declared that this influence occurs “often”, confirming the significant role that physical appearance plays in the daily self-perception of a large proportion of the respondents.

In contrast, 14 people (17.72%) responded that body shape “rarely” influences how they perceive themselves, and 13 people (16.46%) indicated “very rarely”. Five respondents (6.33%) also declared a sporadic influence (“almost never”), while sixteen people (20.25%) considered that body shape did not influence their self-esteem at all (“never”).

In summary, for more than two-thirds of respondents, perceived appearance, including body shape, has a real impact on how they think about themselves, which may be significant in the context of further analyses of self-esteem, motivation to lose weight, and emotional factors related to body weight. For 34.18% of respondents, this aspect played a marginal or no role, which may indicate a greater distance from the pressure of physical appearance.

#### 3.2.4. Dissatisfaction with One’s Body

**Figure 4:**
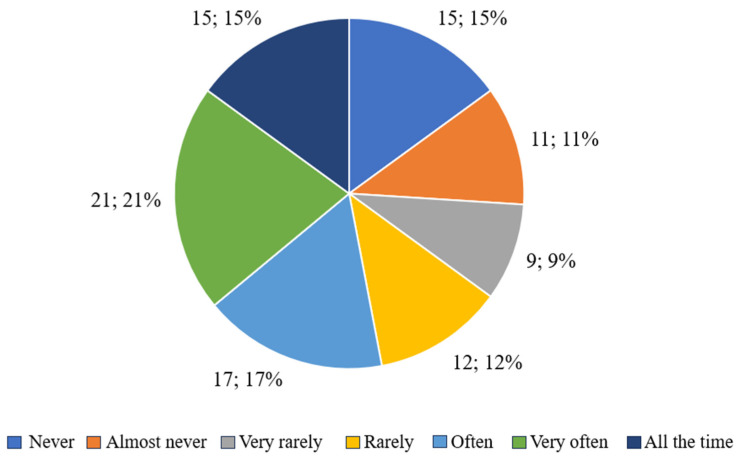
Respondents’ answers to the question “Were you dissatisfied with your body shape?”.

Figure 4 presents the participants’ responses regarding dissatisfaction with their body shape. Over two-thirds of respondents (66.09%) declared that they felt frustration with their body shape to varying degrees, ranging from “often” to “all the time”. The most common responses were “very often” (26.58%) and “all the time” (18.99%), which may indicate strong and regular dissatisfaction with appearance. In contrast, 33.91% of respondents reported that they rarely or never felt such discomfort. These responses may indicate a greater distance from the pressure associated with physical appearance or a more positive self-esteem related to body image.

#### 3.2.5. Physical Activity as a Means of Weight Control

**Figure 5:**
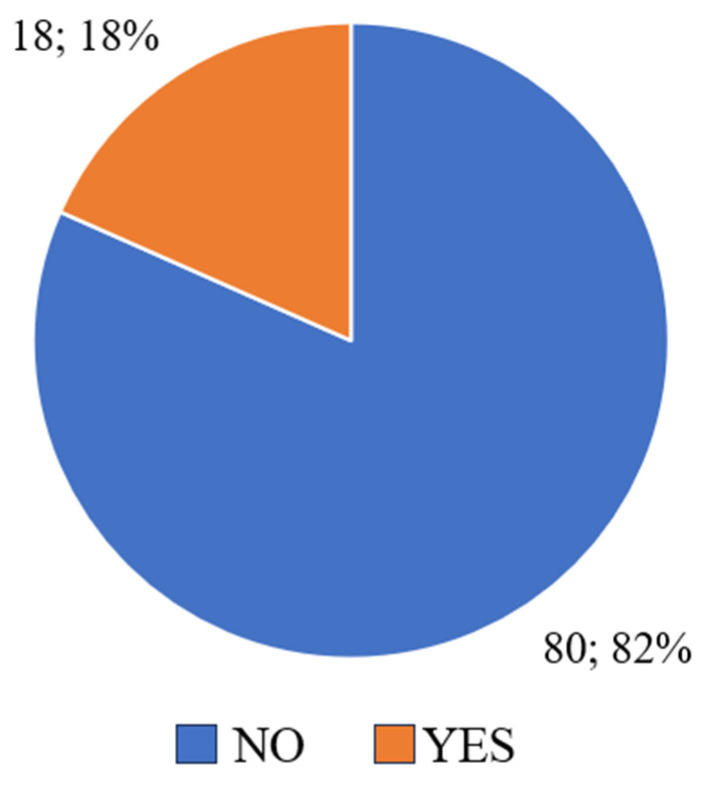
Respondents’ answers to the question “Have you exercised a lot in the last four weeks to avoid gaining weight? If so, how often?”.

Figure 5 shows the responses to the question about physical exercise undertaken to prevent weight gain in the last four weeks. Over half of the respondents (58.58%) declared that they did not exercise intensively, which suggests that physical activity was not their main method of weight control. On the other hand, 41.42% of respondents reported that they exercised regularly to maintain their weight, indicating that for a significant proportion of respondents, exercise was an important part of preventing weight gain.

### 3.3. Psychological Variability Depending on BMI

#### 3.3.1. Respondents’ Answers to the Question “Has Your Body Shape Affected the Way You Think About Yourself?” Taking into Account BMI

An analysis of responses to the question about the impact of body shape on self-perception, taking into account BMI categories, revealed significant differences between the groups (χ^2^ = 17.648; *df* = 18; *p* = 0.0479) (Table 2).

In the underweight group, the most common response was “often” (43%), while among people with normal body weight, the responses “never” and “very rarely” dominated (20% each). In the overweight group, most respondents (38%) admitted that this influence occurs “very often”. In people with grade I and II obesity, the responses were evenly distributed between “often”, “very often,” and “all the time”, which may indicate a strong and persistent influence of body appearance on self-esteem in these groups.

#### 3.3.2. Answers to the Question “Were You Dissatisfied with Your Body Shape?” Taking into Account BMI

The results of the analysis of responses to the question about dissatisfaction with body shape, broken down by BMI categories, showed statistically significant differences between the groups (χ^2^ = 27.43; *df* = 18; *p* = 0.0287).

Underweight respondents most often declared no dissatisfaction (“never”—43%), while in the overweight group, the most common responses were “often” (29%) and “very often” (38%). Among people with stage I and II obesity, the highest percentage of responses was “all the time” (24%), which indicates persistent and intense dissatisfaction with their body shape in these groups (Table 3).

## 4. Discussion

The results of this study confirm the significant impact of overweight and obesity on the psychosocial functioning of children and adolescents. Analysis of the respondents’ answers showed that approximately 48% of those surveyed admitted to eating meals in secret, with 5% declaring that they engaged in this type of behaviour every day for a month. The phenomenon of eating in secret can be seen as a potential indicator of eating disorders, and its increased frequency may indicate the presence of deeper emotional problems, such as shame, guilt, or lack of control over eating. In a study by Kass et al., about one-fifth of adolescents admitted to eating in secret [33]. Other studies have reported the prevalence of this phenomenon ranging from 18.1% to 34% [34,35]. The individuals exhibiting this type of behaviour also showed higher levels of eating-related psychopathology [36]. It is worth noting that children and adolescents are characterized by high emotional sensitivity and susceptibility to environmental influences, which increases the risk of behavioural disorders and difficulties in regulating emotions. Overeating can serve as a mechanism for coping with tension, stress, rejection, or low mood, as evidenced by previous studies [37,38,39,40,41].

Among the participants in this study, approximately 33% of respondents reported daily thoughts about having a flat stomach in the last 28 days, and 65.82% indicated that their body shape significantly affects how they feel about themselves. In addition, more than two-thirds of the respondents felt frustrated with their body shape. These results indicate that physical appearance plays a key role in the process of self-image formation in young people and can significantly affect their mental well-being. Similar results were obtained by Ballarin et al., where as many as 84.6% of obese teenagers were dissatisfied with their bodies [42]. Both previous studies and the results of the presented project confirm that overweight and obese young people have difficulties accepting their appearance. Self-esteem in this group varies according to gender, with girls experiencing lower self-esteem more often than boys [43]. This may be related not only to psychosocial factors but also to biological differences in body composition between sexes. Girls, especially during adolescence, tend to accumulate more body fat, which, combined with increased sensitivity to appearance-related concerns, leads to lower self-esteem and greater body dissatisfaction. Boys, on the other hand, are more focused on muscularity and physical strength, which can result in different patterns of body image concerns. Overweight and obesity are also associated with a lower quality of life, which may result from a lack of acceptance by those around them and feelings of exclusion and powerlessness [44,45]. Children and adolescents struggling with overweight and obesity are prone to behavioural problems, anxiety, behavioural disorders, and depression. Obesity and depression are both public health problems. The relationship is two-way, with the occurrence of one condition increasing the risk of the other [46]. Both girls and boys of school age with a high BMI indicating overweight or obesity tend to be more depressed than their normal-weight peers [47,48,49,50].

Additionally, our study observed clear differences in psychosocial functioning between children (aged 9–12 years) and adolescents (aged 13–18 years). Adolescents exhibited significantly higher levels of body dissatisfaction and stronger internalization of sociocultural ideals of thinness. This group also more frequently engaged in behaviours aimed at weight control, such as restrictive dieting and excessive physical activity, motivated primarily by appearance concerns rather than health reasons. These findings are consistent with the Self-Determination Theory, which suggests that external motivations, such as social and peer pressure related to physical appearance, intensify during adolescence [32]. Exposure to unrealistic beauty standards through social media further exacerbates these issues, leading to emotional distress, lowered self-esteem, and an increased risk of developing eating disorders [31]. In contrast, younger children showed lower levels of body dissatisfaction and engaged in physical activity primarily for enjoyment rather than to control their appearance. It should be emphasized that the observed differences were identified through qualitative data analysis and not through separate statistical analyses comparing age groups. Therefore, the conclusions drawn should be treated as preliminary observations requiring further verification in future studies. Developmental differences highlight the need for age-appropriate intervention strategies. Prevention programs for children should focus on promoting an intuitive approach to eating and building a positive relationship with physical activity, while interventions for adolescents should address sociocultural pressures, support emotional resilience, and develop cognitive-behavioural skills to counteract negative body image [51].

It is worth noting that people with abnormal BMI, who are overweight and obese, have lower self-esteem, which is largely due to dissatisfaction with their own bodies, resulting in feelings of discomfort, for example. In our study, the analysis of responses showed statistically significant differences in body image perception depending on BMI category. In overweight and obese individuals, the influence of body shape on self-perception was clearly stronger and more frequent than in groups with normal body weight or underweight (χ^2^ = 17.648; *p* = 0.0479). Similar relationships were observed in terms of dissatisfaction with appearance—the highest percentage of “all the time” responses was recorded among obese individuals (χ^2^ = 27.43; *p* = 0.0287), confirming the entrenched negative perception of body shape in this group. A study conducted as part of the NutriNet-Santé cohort showed that the relationship between self-esteem and BMI depends on baseline body weight—in people with normal BMI, higher self-esteem was associated with its stability, while in people with grade II and III obesity, higher self-esteem correlated with lower BMI [52].

The results of this study can be interpreted in light of the Self-Determination Theory (SDT), which is one of the most commonly used theoretical approaches in designing interventions and programs aimed at adolescents. According to this theory, individuals have a natural tendency for psychological growth. To support well-being and strengthen intrinsic motivation, it is necessary to meet three key psychological needs: autonomy, competence, and relatedness. Participants with higher BMI levels demonstrated lower intrinsic motivation for physical activity, which may be associated with reduced feelings of competence and autonomy [53].

Moreover, the observed body dissatisfaction may result from social comparisons, in line with the Social Comparison Theory. Adolescents often compare their appearance to idealized images promoted in the media and among their peers, which negatively affects their self-esteem and motivation [54].

The results show that 58.58% of respondents did not engage in intense physical activity to control their body weight, while 41.42% exercised regularly, considering exercise an important part of preventing overweight. Regular physical activity not only helps to control body weight but also contributes to overall improvement in physical and mental health [16]. Strong et al. recommend that children and adolescents engage in moderate to vigorous physical activity for at least 60 min per day. According to the results of the study, children who met these recommendations had significantly lower BMI and better cardiometabolic health indicators [27]. In addition, physically active children have lower levels of obesity, better physical fitness, and lower stress levels compared to children who lead a sedentary lifestyle [14].

Effective prevention of overweight and obesity in children and adolescents requires an integrated approach that combines dietary changes with regular physical activity. Intervention programmes should include both nutrition education and the promotion of physical activity, involving both children and their families. Joint efforts can lead to lasting lifestyle changes that will promote the healthy development of children and adolescents [55,56]. Encouraging daily activity and planning regular sports activities, such as dance classes or group training, helps to introduce structure and routine, which is particularly important for young people [57]. In addition, regular physical activity leads to improved cardiovascular health, better weight control, stronger muscles and bones, and has a positive impact on mental health [58,59,60].

Other researchers have pointed to the need to create public spaces that promote an active lifestyle and to involve local communities in promoting physical activity among young people [61]. It should be emphasized that physical activity in early childhood is crucial for preventing excessive weight gain. Kindergartens and family homes should create conditions conducive to physical activity, and parents should actively participate in encouraging their children to engage in physical play. The findings suggest that early habits related to physical activity can have a long-term impact on health and reduce the risk of obesity [30,62]. Studies conducted in Poland also confirm that interventions combining health education with practical activity yield the best results [29,63]. Other researchers have demonstrated the effectiveness of school programmes promoting physical activity, especially in reducing sedentary lifestyles [64,65]. The results of the study suggest a significant association between overweight or obesity and negative body image, low self-esteem, and limited physical activity among children and adolescents, based on observed group-level differences.

Despite the significant results obtained, this study has some limitations. Firstly, the use of a self-report questionnaire involves the risk of cognitive errors and response bias, resulting, among other things, from the desire to present oneself in a favourable light (the so-called social approval effect). Secondly, the cross-sectional nature of this study prevents causal conclusions from being drawn. In addition, the research sample was limited geographically and demographically, which may limit the generalizability of the results to the entire population of children and adolescents in Poland. Finally, environmental and family factors were not analysed in depth, which could have provided a broader context for the identified phenomena. Although preliminary reliability tests (e.g., Cronbach’s alpha) were conducted, the applied questionnaire has not undergone formal validation. However, one of the strengths of this study is its comprehensive approach to the issue of overweight and obesity among children and adolescents, covering both psychological and behavioural aspects, such as body image, self-esteem, and physical activity. In addition, the use of standardized measurement tools increases the reliability and comparability of the results with other studies in this field.

Finally, it is worth noting that a key strength of this study is the simultaneous consideration of both somatic and psychosocial variables, which allows for a comprehensive approach to the problem. Moreover, by including different age groups, this study enables an analysis of psychosocial changes across various stages of development. Although this study provided many important insights into the emotional state of young people aged 9 to 18, there is a need for further research to obtain a more complete understanding of the situation. Longitudinal studies would make it possible to assess the sustainability of the observed effects, particularly in the context of educational interventions. The results of this study may have significant practical implications, especially in the field of health education and prevention. In the context of health policy, the obtained findings may serve as a basis for developing support programs for parents and teachers that promote healthy habits in children. Additionally, it should be emphasized that this study did not analyse differences between boys and girls across various age groups, which should be addressed in future research to better understand the distinct developmental and behavioural factors.

## 5. Conclusions

This study highlights the complexity of the problems faced by overweight and obese children and adolescents. Both body image and self-perception are closely related to body weight, which manifests itself, among other things, in frequent thoughts about the ideal body shape, feelings of frustration, and low self-esteem. Eating meals in secret may indicate developing emotional difficulties and eating disorders that require special attention and psychological support. In addition, the low level of physical activity in the study group may indicate a need to intensify educational and promotional activities promoting a healthy lifestyle from an early age. In the context of the results obtained, it is necessary to implement comprehensive prevention programmes which, in addition to dietary aspects, will also include work on a positive body image and a healthy relationship with food. These programs should be coordinated by national and local public health and education authorities, with implementation in schools supported by health professionals and NGOs. Funding could be provided through local and national budgets and European Union resources. Moreover, this study did not assess differences in overweight, obesity, and body image perception between children and adolescents or between girls and boys, which should be addressed in future research.

## Figures and Tables

**Table 1:** BMI (Body Mass Index) of respondents.

BMI		Health Resort Hospital	Clinical Hospital	Secondary School
starvation	<16.0	0	0	0
emaciation	16.0–16.9	0	0	0
underweight	17.0–18.5	0	1	6
normal weight	18.5–24.9	7	9	21
overweight	25.0–29.9	7	10	4
obesity class I	30.0–34.9	11	6	0
obesity class II	35.0–39.9	5	3	0
obesity class III	≥40	0	0	0

**Table 2:** Respondents’ answers to the question “Has your body shape affected the way you think about yourself?” taking into account BMI.

Has Your Body Shape Affected the Way You Think About Yourself?	BMI							
	Underweight		Normal Weight	Overweight	Obesity Class I		Obesity Class II	
	n	%	n	%	n	%	n	%
never	1	14%	9	20%	1	5%	2	12%
almost never	1	14%	1	2%	1	5%	2	12%
very rarely	1	14%	9	20%	1	5%	2	12%
rarely	1	14%	5	11%	4	19%	2	12%
often	3	43%	7	16%	4	19%	3	18%
very often	0	0%	7	16%	8	38%	3	18%
all the time	0	0%	6	14%	2	10%	3	18%
Chi-square	χ^2^ = 17.648		*df* = 18		*p* = 0.0479			

**Table 3:** Respondents’ answers to the question “Were you dissatisfied with your body shape?” taking into account BMI.

Have You Been Unhappy with Your Body Shape?	BMI							
	Underweight		Normal Weight	Overweight	Obesity Class I		Obesity Class II	
	n	%	n	%	n	%	n	%
never	3	43%	9	20%	1	5%	1	6%
almost never	0	0%	8	18%	1	5%	1	6%
very rarely	2	29%	3	7%	0	0%	3	18%
rarely	0	0%	5	11%	2	10%	3	18%
often	1	14%	8	18%	6	29%	2	12%
very often	1	14%	5	11%	8	38%	3	18%
all the time	0	0%	6	14%	3	14%	4	24%
Chi-square	χ^2^ = 27.43		*df* = 18		*p* = 0.0287			

## Data Availability

All data and materials are included in the manuscript.
